# Physical pain-suicidality association in all ages: a complete and updated meta-analysis

**DOI:** 10.1192/j.eurpsy.2022.829

**Published:** 2022-09-01

**Authors:** M. Rignanese, E. Salmè, M. De Filippi, F. Madeddu, M. De Prisco, M. Fornaro, R. Calati

**Affiliations:** 1 Università degli Studi di Milano-Bicocca, Psychology, Milan, Italy; 2 University of Milano-Bicocca, Psychology, Milan, Italy; 3 University School of Medicine Federico II, Section Of Psychiatry - Department Of Neuroscience, Reproductive Sciences And Dentistry, Naples, Italy; 4 University of Milan-Bicocca, Department Of Psychology, Milan, Italy; 5 Nîmes University Hospital, Department Of Adult Psychiatry, Nîmes, France

**Keywords:** suicidal behaviours, suicidal thoughts, physical pain, meta-analysis

## Abstract

**Introduction:**

This work represents the continuation of the studies presented in two e-posters during the EPA 2021 conference (De Filippi et al., 2021; Rignanese et al., 2021), which addressed the physical pain-suicidality association (k=44 studies).

**Objectives:**

The aim of this meta-analysis is to provide an update of those studies, integrating data relating to adolescents, adults, and olders.

**Methods:**

We started with the analysis of three papers, in particular a meta-analysis (Calati et al., 2015) and two systematic reviews (Hinze et al., 2019; Santos et al., 2019). After searching on Pubmed (until September 2020), data were extracted from articles comparing the rates of current and lifetime suicidal thoughts and behaviours (death wish, suicidal ideation, suicidal planning, suicide attempt and suicide death: DW, SI, SP, SA, and SD) in adolescents, adults, and olders with any type of physical pain and in individuals who did not report this condition. Data were analysed using Comprehensive Meta-Analysis Software (CMA) version 2.

**Results:**

67 studies were included, of which 16 on adolescents, 29 on adults, 16 on olders, and 6 on mixed ages. Although quite high between-study heterogeneity was detected in most analyses, results suggested that individuals with physical pain are more likely to report any form of suicidal outcome if compared to those not affected by pain.

**Conclusions:**

Collected data are therefore in line with previous literature on this topic, which considered physical pain an extremely predictive risk factor for suicidal thoughts and behaviours. However, further research on this topic would be extremely useful.

**Disclosure:**

No significant relationships.

